# Long term tailored implementation of structured “TREAT” journal clubs in allied health: a hybrid effectiveness-implementation study

**DOI:** 10.1186/s12909-022-03333-7

**Published:** 2022-04-22

**Authors:** Rachel Wenke, Jodie Wiseman, Caitlin Brandenburg, Paulina Stehlik, Ian Hughes, Katherine Richards, Sharon Mickan

**Affiliations:** 1 Gold Coast Hospital and Health Service, 1 Hospital Boulevard, Southport Queensland, 4215 Australia; 2 Faculty of Health Sciences and Medicine, Bond University, Gold Coast, Queensland Australia; 3 School of Health Sciences and Social Work, Griffith University, Gold Coast Campus, Gold Coast, Queensland 4222 Australia; 4 Occupational Therapy, Faculty of Health Sciences and Medicine, Bond University, Gold Coast, Queensland Australia; 5 Institute for Evidence-Based Healthcare, Bond University, Gold Coast, Queensland Australia

**Keywords:** Journal clubs, Evidence-based practice, Allied health, Implementation

## Abstract

**Background:**

Allied Health Professionals (AHPs) commonly use journal clubs (JCs) to support Evidence-Based Practice (EBP). There is however little research regarding implementing and sustaining JCs in the long term, and their impact on EBP use and skills in AHPs. This study investigated the impact of implementing a structured JC format, called “TREAT” (previously only investigated across 6 sessions), over a longer period of 16 sessions for AHPs in a public health service. The study also investigated AHP’s attendance, adherence, satisfaction and barriers and enablers to implementing the format.

**Methods:**

A mixed methods hybrid-effectiveness implementation design was employed, guided by the Knowledge-to-Action cycle. EBP skills, confidence, use, and attitudes were assessed (Adapted Fresno Test, EBPQ, tailored journal club culture questionnaire) at baseline, and after 10 and 16-monthly sessions. Satisfaction and impact on clinical practice were explored using questionnaires at 10 and 16-months, with free-form responses identifying enablers and barriers to EBP culture and implementation. Data on attendance and adherence to the TREAT format were also collected.

**Results:**

Six JCs comprising a total of 132 unique participants from seven Allied Health professions were assessed across three time points. EBP skills improved on the Adapted Fresno Test after 10-monthly (6.6 points: 95% CI, 0.43 to 12.7) and 16-monthly sessions (7.8 points, 95% CI, 0.85 to 14.7), and on self-reported total EBPQ ratings of confidence at 10-months (4.9 points: 95% CI, 2.2 to 7.5) and 16-months (5.7 points: 95% CI 2.7 to 8.7). Of 132 AHPs, 88 reported adopting new treatments/resources and 64 reported updating clinical procedures. Mean attendance was 5.7 sessions (SD = 3.8). Adherence to TREAT components in each session was 86% (95% CI, 83% to 89%). Most participants recommended the format and reported a desire to continue. Enablers to the JC included using clinically relevant topics and active participation while reported barriers included limited time to prepare.

**Conclusions:**

Despite variable attendance, TREAT JCs can continue to be implemented within a service for 16 monthly-sessions, and may contribute to improved EBP skills and confidence and changes in clinical practice over time. Tailoring of implementation strategies was shown to be important to address local enablers and barriers.

**Supplementary Information:**

The online version contains supplementary material available at 10.1186/s12909-022-03333-7.

## Background

Evidence-Based Practice (EBP) is recognised as a foundational competency for safe and effective practice by health professionals [1]. While many health professionals graduate from university feeling moderately confident in their EBP skills, this confidence declines in the first five years of clinical practice [2], highlighting the need for workplace-based interventions to facilitate ongoing use of EBP. One method for integrating EBP into everyday clinical care is the use of journal clubs (JCs) [3, 4]. JCs involve individuals meeting to evaluate and discuss research evidence and its implications for clinical practice [5, 6] thus enabling currency of clinical practice, and the learning of EBP skills [7].

While JCs are commonly used by health professionals, further knowledge is required to optimise their implementation and effectiveness within clinical settings [7]. Additionally, JCs rarely incorporate evidence-informed components that support sustained implementation or use principles of adult learning to target the needs of clinician learners [8, 9]. To address some of these shortcomings, a structured JC format- TREAT (Tailoring Research Evidence and Theory) was developed and tested in an RCT [5]. TREAT uses 11 evidence based components to enhance a JC’s effectiveness tailored to the allied health setting [5, 9, 10]. While health professionals have reported significantly greater satisfaction with the TREAT format, there were no significant improvements in EBP skills, attitudes, or knowledge and/or practice, compared to the standard format after 6 months participation [5]. We proposed that the 6-month implementation period was not long enough for significant changes to be identified. As other research on structured allied health JCs has also been within a 6-month period [5, 11], further investigation is needed to explore the effectiveness of JCs over a longer duration.

It is important to understand what factors influence JC sustainability. A follow up qualitative study synthesised a range of implementation strategies that may enhance opportunity, motivation and capability for sustainability of TREAT JCs within local contexts [12]. As JCs function within their own unique culture and context, tailoring of strategies to each JC is likely required for successful long-term implementation. Specific tailoring of how to best implement TREAT JCs in each individual team’s local context has however not yet been investigated.

### Objectives

The primary aim was to identify which tailored implementation strategies were most effective in promoting AHPs JC attendance, culture, and satisfaction over a longer term (16-month) implementation across different JC contexts. Secondary aims were to investigate the long-term (16-months) impact of implementing the TREAT JC format with implementation strategies specifically tailored to each JC’s local context on AHPs’ EBP skills, confidence, use and changes in clinical practice; and JC satisfaction, attendance, and adherence; and identifying barriers and enablers to its implementation. This manuscript presents the results of these secondary aims, while the primary aim will be reported as part of a realist evaluation (currently in preparation).

## Methods

### Study design

A hybrid implementation-effectiveness study (type 3) design [13] using a mixed methods approach guided by the Knowledge to Action (KTA) cycle [14] was conducted. Data was collected at baseline, and after 10 and 16-monthly scheduled TREAT JC sessions. Due to the primary implementation focus of this trial and secondary effectiveness focus, all participants received the TREAT JC format, with reporting guided by the EQUATOR Standards for reporting implementation studies [15].

### Participants

Participants were recruited from any Allied Health Professional (AHP) JC (both new and existing) within a single non-metropolitan Australian public health service which consisted of two main hospitals and several community centres. Manager’s support was gained prior to consenting JC participants. Research mentors who supported the JCs were invited to participate in an audit of their support activities.

### Ethical Approval and Consent to Participate

Ethical approval for the study was sought by the Gold Coast Hospital and Health Service Human Research Ethic Committee (HREC/15/QGC/310) and all participants provided written informed consent to participate. All methods were performed in accordance with the relevant guidelines and regulations.

### Procedure

Table 1 provides an overview of the study procedure including assessment time points and alignment with steps of the KTA cycle.

**Table 1:** Overview of study procedure matched to the KTA cycle

Phase	Step in KTA cycle	Activity
**Pre implementation**	Adapt knowledge to local context Assessing readiness/barriers to knowledge use	Pre-Assessment (Baseline) -Topic selection - First four JC topics brainstormed and chosen as a group
	Selection of implementation strategies	30 min discussion with JC clinician facilitators to select implementation strategies based on results of Journal Club Readiness and Culture questionnaire results
**Journal club implementation**	Monitoring knowledge use	JC Sessions 1 & 2: Researcher facilitates JC Session 3 & 4: Clinician co-facilitates with researcher JC sessions 5–10- Clinician facilitates Measure adherence TREAT format throughout implementation
	Evaluate outcomes	Assessment after 10 JC sessions (approx. 10-months post implementation) Clinician focus group
	Sustain knowledge use	Assessment after 16 JC sessions (approx. 16-months post implementation) Clinician focus group

#### Pre-implementation

Prior to implementing the JCs, at least two clinicians from each JC volunteered to be facilitators and attended a four-hour in-house EBP workshop run by Bond University. An AHP researcher was also allocated to be the research mentor for each JC, who received their own training (approximately 4 h of face to face and reading). Participants in each JC then completed a baseline assessment and voted on topics for their upcoming JCs during a 60 min session facilitated by the research mentor.

#### Identification of Implementation Strategies

Approximately 1 week after the initial session, the research mentor met with the JC clinician facilitators plus 1–2 other JC members to discuss a summary of the baseline assessment results and co-design a tailored implementation plan based on a list of potential implementation strategies (see Supplementary file 1 for the list and see Supplementary File 2 for an example implementation plan).

### Intervention

The TREAT JCs ran for one hour, once a month over approximately 16 months, with flexibility and rescheduling required over holiday and busy service periods. Key components of the TREAT structure included: group appraisal of articles using the Critical Appraisals Skills Programme (CASP) structured appraisal tools [16], discussion of the evidence in the context of clinical practice, and having set facilitator, presenter and scribe roles in the JC. An example session plan is found in Supplementary File 3 with further details published previously [5]. To support clinician facilitator’s confidence and capability, the first two TREAT JC sessions were planned to be facilitated by the research mentor, followed by two sessions of co-facilitation by the research mentor and the clinician facilitator, then two sessions led by the clinician facilitator (with the research mentor still present). The research mentor remained contactable for support between sessions throughout the duration of the 16-month implementation.

### Outcome measures

#### EBP proficiency

EBP Questionnaire (EBPQ): This questionnaire involves 24-items asking participants to self-rate their (1) attitudes towards, (2) confidence in, and (3) use of EBP on three separate subscales [17].

Adapted Fresno Test [18]: Is an objective measure of EBP skills comprising 7 questions related to one of four clinical scenarios. The test was scored by a researcher (JW), who was blinded to the participant’s JC and the time point at which the test was completed. Intra-rater reliability of the Adapted Fresno has been found to be high [19].

Journal club readiness and culture (JCC) questionnaire*:*

Participants rated 11 items on a 5-point Likert scale, with potential responses ranging from strongly disagree to strongly agree. An open-ended questionnaire regarding barriers and facilitators to EBP and running of JCs within their teams was completed.

#### Influence of journal club on clinical practice

A tailored questionnaire which included questions for each JC session regarding attendance, relevance of the appraised article, and a 5-point Likert scale rating whether the evidence from the appraised article led to changes in practice or confirmed their current practice. If a change in practice was indicated, participants identified what change was made from a list of multiple-choice options (e.g., updated clinical guideline, adopted new treatment strategy).

#### Fidelity measures

Clinician facilitators completed an audit questionnaire after every JC session to identify which key components of the TREAT format were adhered to, with space for additional comments and reflective questions. After 10-monthly sessions, research mentors were asked to complete a custom multiple-choice questionnaire to indicate what level of support they provided within each JC session. Multiple choice options ranged from a maximal level of support (i.e., facilitating the session) to no support provided (i.e., not attending the session and not providing any active support), with an option for free form comments.

### Data analysis

Frequencies and percentages were calculated for demographic information (Table 1). Means were estimated with standard errors (SE) for EBPQ, Adapted Fresno Total, and JCC questionnaire scores at baseline, 10-months and 16-months. Sample size was estimated based on comparing a difference in mean EBP-Q between two time points. For a simple two-sided paired t-test to detect a change of 6 points on the EBP-Q with 80% power at a significance level of 0.05 it was determined that a sample of 28 participants was needed (G*Power 3.1). The EBP-Q was chosen as the primary outcome due to its wide use. In the absence of literature, the study authors considered a change of 6 points on the EBP-Q confidence scale (i.e., self-reported knowledge) to be the minimum clinically important change that was unlikely due to test variability. Within group changes for quantitative measures (i.e., Adapted Fresno, EBPQ, Likert scale questionnaires) between time points were initially assessed with paired t-tests. Subsequently, mixed effects regression models were used with the JC and individual participant identity considered as random effect levels. Time-point was considered the variable of interest and the effects from baseline to 10-months and baseline to 16-months reported. Prior EBP training, gender, age group and total JC sessions attended were considered as fixed effect covariates. Where a covariate effect was seen with *p* < 0.05 it was included in the model. Adjusted time-point effects are reported.

Qualitative content analyses were used for open-ended questionnaire responses for the 10-month and 16-month data. In this approach, one of the authors (JW or CB) coded meaning units into categories and subcategories [20] using NVivo software. These were then checked by a second author (RW). Once all data was analysed, qualitative and quantitative data were interpreted jointly.

## Results

A total of 132 unique clinician participants consented to take part in the study across six JCs. Demographic information of participants is found in Table 2. Most participants were female (*n* = 111, 84%) with 2 to 10 years of clinical experience (*n* = 79, 59%), and were currently working in the inpatient acute setting (*n* = 67, 51.5%). Of the 132 participants, 79 (61.2%) from six JCs completed the baseline assessment, 63 (48.8%) from six JCs completed the 10-month assessment and 47 (49%) from four JCs completed the 16-month assessment. Two of the JCs did not participate in the 16-month assessment due to the COVID-19 pandemic. Four research mentors completed the support audit (median post-doctorate experience was 4.5 years, range 4 to 11 years).

**Table 2:** Demographics of participants

Demographics	N (%) 132 unique participants
*Gender*	
Male	21 (15.9%)
Female	111 (84.1%)
*Age (years)*	
20–29	71 (53.8%)
30–39	41 (31.1%)
40–49	16 (12.1%)
50–59	4 (3.0%)
*Profession*	
Dietician	3 (2.3%)
Pharmacist	18 (13.6%)
Dentist	18 (13.6%)
Psychologist	1 (0.8%)
Occupational Therapist	45 (34.1%)
Speech Pathologist	45 (34.1%)
Physiotherapist	1 (0.8%)
Allied Health Assistant	1 (0.8%)
*Setting*	
Community setting	17 (13.1%)
Hospital Outpatient	7 (5.4%)
Inpatient Acute	67 (51.5%)
Multiple	28 (21.5%)
Clinical Education	3 (2.3%)
Inpatient Mental health	1 (0.8%)
*Clinical experience (years)*	
Less than 2	26 (19.4%)
2–5	41 (30.6%)
6–10	38 (28.4%)
9–15	13 (9.7%)
Greater than 15	16 (11.9%)
*Higher Research Degrees*	
None	46 (60.5%)
Masters of Research	2 (2.6%)
PhD	1 (1.3%)
Honours	12 (15.8%)
Masters—Other	7 (9.2%)
Graduate Diploma	5 (6.6%)
Post Graduate Certificate	3 (4.0%)
*Have attended EBP Training*	53 (40.1%)
*Number of journal clubs attended* Mean (SD)	5.72 (3.83)
*Completed Assessment at baseline*	79 (61.24%)
*Completed Assessment 10-months*	63 (48.84%)
*Completed Assessment 16-months*	47 (48.96%)

### EBPQ and Adapted Fresno Test

Improvements in EBP confidence were observed at 10- and 16-months on the EBPQ (Table 3). Minimal changes were apparent in EBP attitudes and EBP use across time points. On the Adapted Fresno Test EBP skills were found to be improved at 10 and 16-months (Table 3).

**Table 3:** EBPQ, Adapted Fresno Total, and Journal Club Culture Questionnaire scores at baseline, 10-months and 16-months: Model adjusted Means and Mean Differences

	Baseline/Pre Mean (SE)	10 month Mean (SE)	16-months Mean (SE)	Pre-10 month Mean Difference and 95% CI, *p*-value	Pre-16 month Mean Difference and 95% CI, *p*-value
***EBPQ & Adapted Fresno***					
EBP use (Q1 total, max score = 42)	23.4 (1.2)	25.5 (1.3)	24.3 (1.3)	2.2 (0.4, 4.1) 0.015	0.9 (-1.1, 2.9) 0.4
EBP attitudes (Q2 total, max score = 28)	21.7 (0.4)	21.2 (0.5)	21.8 (0.5)	-0.41 (-1.3, 0.5) 0.390	0.2 (-0.8, 1.2) 0.680
EBP confidence (Q3 total, max score = 98)	56.9 (1.8)	61.8 (1.9)	62.6 (2.0)	4.9 (2.2, 7.5) 0.000	5.7 (2.7, 8.7) 0.000
EBP skills (Adapted Fresno Test) Total (max score = 168)	70.4 (5.6)	80.0 (5.7)	78.2 (5.9)	6.6 (0.4, 12.7) 0.036	7.8 (0.9, 14.7) 0.028
***JCC Questionnaire (maximum score of 5* = *strong agreement)***					
Use of EBP valued by team	4.4 (0.1)	4.6 (0.1)	4.6(0.1)	0.1(-0.1, 0.2) 0.220	0.1 (-0.03, 0.3) 0.100
Manager expects attendance	4.0 (0.3)	4.3 (0.3)	4.2 (0.3)	0.26 (0.04, 0.5) 0.020	0.2 (-0.1, 0.4) 0.160
Sense of JC ownership	3.9 (0.2)	3.7 (0.2)	4.0 (0.2)	-0.2 (-0.4, 0.1) 0.160	0.1 (-0.1, 0.3) 0.420
JC is applicable to practice	4.4 (0.2)	4.1 (0.2)	4.1 (0.2)	-0.3 (-0.5, -0.1) 0.002	-0.3 (-0.5, -0.1) 0.004
JC attendance is a priority	4.0 (0.3)	3.8 (0.3)	3.7 (0.3)	-0.1 (-0.4, 0.1) 0.190	-0.3 (-0.5, 0.0) 0.035
JC is of benefit to me	4.3 (0.2)	4.1 (0.2)	4.0 (0.2)	-0.12(-0.4, -0.0) 0.050	-0.3 (-0.5, -0.0) 0.020
Group participation valuable part of JC	4.4 (0.1)	4.5 (0.1)	4.6 (0.1)	0.1 (-0.1, 0.2) 0.500	0.2 (-0.0, 0.35) 0.100
Sharing of knowledge and skills within team	3.7 (0.2)	3.9 (0.2)	4.0 (0.2)	0.2 (-0.1, 0.4) 0.2	0.3 (0.1, 0.6) 0.010
Access to expertise and resources outside team	3.5 (0.1)	3.8 (0.1)	4.0 (0.1)	0.3 (0.1, 0.5) 0.020	0.6 (0.3, 0.8) 0.000
Think JC should continue	N/A	3.8 (0.2	3.7 (0.2)	-0.0 (-0.3, 0.2) *p* = 0.870 (10 – 16-months)	
Recommend JC to others	N/A	3.9 (0.2)	4.0 (0.2)	0.1 (-0.2, 0.3) *p* = 0.607 (10 – 16-months)	

*N.B: Mean* model-based estimate of mean score, *SE* standard error for model-based estimate

### Journal club culture questionnaire

Clinician ratings of their manager’s expectation for them to attend JC and clinicians’ access to expertise and resources outside the team were found to improve at 10-months compared to baseline, with the latter continuing to improve 16-months following JC participation (see Table 3). An increase at 16-months compared to baseline was also found for information sharing within the team. At the 10-month and 16-month time points, the ratings of agreement to wanting the JC to continue and recommending it to others were between 3.7–4.0 out of 5.

When covariates were taken into consideration, prior EBP training was found to have a positive effect on the Adapted Fresno total scores, with adjusted changes from baseline to 10 months being 6.4 (95% CI: 0.23, 12.5; *p* = 0.042) and baseline to 16 months 7.8 points (95% CI: 1.09, 14.9; *p* = 0.023). Gender and total attendance were found to have a significant effect on the group participation item of the JCC questionnaire leaving a negligible effect of time-point at 10 months; 0.01 (95% CI: -0.17, 0.19, *p* = 0.90) and 16 months; 0.11 (95% CI -0.06, 0.29, *p* = 0.37) compared to baseline.

### Qualitative responses of journal club and culture questionnaire

Five categories were identified in the questionnaire responses: 1) enablers and 2) barriers to EBP culture, 3) enablers and 4) barriers to JC; as well as 5) suggestions for improvement. Most commonly reported categories and subcategories are shown in Table 4. In general, responses did not differ across time points. Lack of preparation due to time constraints and variable attendance were reported as barriers to JC at the 10-month time point but were not reported at the 16-month time point. The most commonly reported enablers to EBP culture were team educational opportunities (with “*Journal club*” being the most frequently reported of these opportunities); and “Collaborators and People Resources”. The most predominant barrier was “time and competing caseload demands and difficulties implementing EBP as described as “*possible lack of confidence in integrating EBP into clinical practice if there is a change required*” (P027).

**Table 4:** Responses for JCC open ended questions – 10-months

**Categories and subcategories**	No. of mentions 10-months	No. of mention s 16-months
**1) Enablers of EBP culture**		
*Team educational opportunities*		
Professional Development in clinical skills/ in-services	12	15
Individual learning time	9	1
Quality projects and portfolios	7	6
Regular supervision	8	9
Encouragement of CPD courses	6	6
Journal club	19	29
Team discussions EBP literature and clinical cases	12	4
*Collaborators & People resources (e.g., EBP champions, Research Fellows)*	14	18
*Supportive workplace culture that values EBP*	15	10
Supportive managers and seniors	7	9
Protected time	8	1
It's an expectation	7	4
*Accessible resources*	6	5
**2) Barriers of EBP culture**		
*Time and caseload demands*	40	37
*Difficulties with EBP implementation*	4	14
*Personal and team reduced knowledge & skills*	5	4
*Type and quality of research*	3	2
*Staffing issues*	4	1
Lack of EBP value or commitment	1	4
**3) Enablers of Journal Club**		
*Structure*		
Having an academic present or support available	11	8
Set regular times	6	4
Protected time	6	9
Clinically relevant topics	11	19
Early circulation of articles	2	10
Preparation of presenter and facilitator	5	4
Specific roles allocated and shared	6	9
*Supportive team factors*	16	9
*Supportive leadership, managers and seniors*	6	7
*Research and clinical knowledge*	7	3
*Attendance and active participation*	19	12
**4) Barriers of Journal Club**		
*General time constraints*	8	20
*Irrelevant topics*	7	12
*Personal factors such as fatigue, motivation, stress*	6	5
*Variable attendance*	5	0
Staffing impacting attendance	11	2
*Lack of active participation or preparedness*	7	11
*Reduced knowledge, skills & confidence*	10	6
*Lack of preparation generally due to time constraints*	22	0
**5) Suggestions for improvement**		
*Increased education and support*	19	7
*Structure changes*	14	18
*Scheduling changes*	7	3
*Changes to preparedness*	7	3
*Topic selection suggestions*	3	11

Enablers to JC related to structural components of the format included choosing clinically relevant topics, “*having a team consensus on choosing articles relevant to the group” (P119)* and active participation from the group, with one clinician commenting, *“Group participation is important for the success of journal club”* (P063). Supportive team factors were also commonly reported as enablers, including “*Sharing between all team members* “(P106) and “*Open mindedness and respect of differing opinions*” (P128). Conversely, barriers to JC included topics not being relevant, lack of active participation or preparedness and general time constraints, for example *“time constraints in preparing for JC i.e. pre-reading article.” (P005).*

### Influence on clinical practice

Across the 16 sessions, 64 clinicians reported updating guidelines, processes, or pathways as a result of JC participation, 88 clinicians reported adopting new treatment strategies or resources, 30 clinicians reported starting new quality projects and 6 clinicians reported starting new research projects. One clinician reported discontinuing a current practice due to lack of evidence as appraised in a JC session (See Supplementary file 4).

### Treatment fidelity

**Fig. 1:**
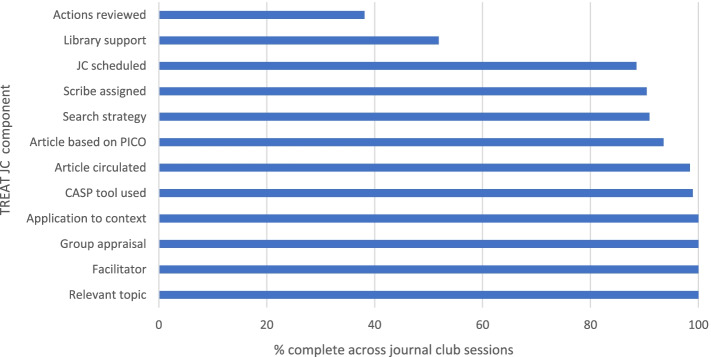
Percentage adherence of TREAT journal club components across sessions

As shown in Fig. 1, components reported to have the highest adherence (100%) were having a relevant topic, a facilitator present, group appraisal and application to clinical context discussed. Items with lowest adherence were reviewing actions from the minutes (38.1%) and seeking library support (51.8%). The first two sessions were facilitated by the research mentor (see Supplementary 5). Although the level of support varied across JCs, a general pattern of reduced support as the sessions progressed was evident in all JCs.

## Discussion

Clinicians who participated in at least 10 months of tailored TREAT JCs improved their EBP skills, reported greater EBP confidence, and EBP-linked changes to clinical practice. These improvements were not observed over 6 months in the same structured JCs [5]. Overall clinician satisfaction and adherence to the TREAT JC was reasonably high, indicating the feasibility of implementing a JC using the TREAT format for up to a 16-month period.

This is the first study to explore longer term impacts of structured allied health JCs. A recent systematic review highlighted that the majority of evidence to date has been from the medical professions [7] and recommended the need for further research to support the effectiveness of JCs in improving knowledge, attitudes and EBP implementation for all health professionals. Improved EBP skills identified on the Adapted Fresno Test and improved self-reported confidence on the EBPQ suggest that JCs may actually increase levels of EBP skills in AHPs. This is particularly important, as EBP skills have been reported to degrade with increasing time in clinical work [2].

The improved EBP skills and confidence occurred with an average attendance of 6 out of a total of 16 sessions. Conversely, an average attendance of 4 sessions out of a possible 6 did not report confidence and skill changes in an earlier study [5]. This may suggest that there may be a threshold number of JC sessions (i.e., 6 sessions) to see changes in EBP confidence and skills, which may also be influenced by tailoring implementation strategies for each JC’s specific context [5]. The finding that attendance as a covariate didn’t influence the majority of outcome measures may further suggest that rather than attendance having an additive or linear effect on EBP proficiency with each session, a more complex interaction type effect may be occurring whereby there may be no effect for the first few sessions and then a threshold effect which may then plateau. It is also possible that despite some participants not attending sessions, they may have still had discussions about the JC session that they missed, which may have facilitated instilling EBP culture and knowledge to the broader group.

The JCC questionnaire and qualitative clinicians ‘ reports identified that access to expertise and resources outside the team and sharing of knowledge within the team improved as the JC continued across 10 and 16 sessions. In particular, supports including EBP champions and research fellows, access to resources, and a supportive team culture were enablers to a positive research culture and/or journal club implementation. These are likely to be important factors in sustaining TREAT JCs [12]. These factors, together, with a tapered approach to reducing formal research mentor support may have promoted clinician facilitators’ independence in running the JC. This positive culture may have been reciprocatively sustained by the ongoing participation and outcomes of the JC, as reflected in ratings of EBP value on the JCC being consistently high over time.

Over 16 months, despite recognised barriers to applying evidence in clinical practice, a large proportion of clinicians self-reported that they adopted new treatment strategies and changed clinical processes following participation in JC sessions. This finding adds to the evidence base demonstrating the impact and value of JCs on real-world clinical practice, something which has had little exploration previously [3]. It is recognised that evidence for many areas of AHP practice are still emerging [21] and therefore may not be able to be readily applied in practice. This barrier may partially account for the downtrend in clinicians’ perspectives of the benefit of the JC on the JCC questionnaire which although remaining high, gradually reduced over time as perhaps some clinicians became more aware of the lack of evidence to change practice in their area.

### Limitations and future directions

Due to a high turnover of participants, fewer data points at 10- and 16-month assessments were identified compared to baseline. Variable attendance and high turnover has been reported in previous JC research, and many clinicians rotate between teams in large departments [5, 22]. In addition, while four of the six JCs in the present study sustained the format for 16 sessions and anecdotally have continued to use the JC several months after the study completion, two JCs were unable to continue the format due to unforeseen service changes related to the COVID-19 pandemic.

Although the study used an objective Adapted Fresno test, responses from the self-report measures (i.e., EBPQ and JCC) could be influenced by social desirability bias. Clinical changes were also self-reported by clinicians and similar changes may have been counted more than once by different participants, therefore future research may benefit from auditing these changes to further substantiate and describe them. Generalisability of the results may be limited by the fact that participants were all from one hospital and health service setting. It is therefore unclear what the impacts of TREAT JCs may be across other contexts within Australia and internationally including primary health care and private practice. It should also be acknowledged that use of the pre-post study design has inherent biases when evaluating effectiveness outcomes and it is possible that improvements in outcome measures reported may be the result of factors other than JC participation that were not accounted for in the study. While an initial RCT evaluating the effectiveness of TREAT JCs has been published [5], future research should investigate the use of TREAT JCs in other contexts and professions where possible using a controlled study design. Further exploration regarding how to enhance the efficiency of the format and handover of tasks and roles to new staff should also be undertaken, as well as determining the optimal frequency of the JC sessions required to have a beneficial effect. Integration of ongoing training particularly for new staff joining the team in the JC format and basic EBP processes is also indicated. Many of these areas will be explored in future research evaluating a web-based system (wwww.treatjournalclubs.com) for sharing resources and tools to support TREAT JC implementation across contexts and professions internationally.

### Clinical implications

This study highlights that a structured JC format can be implemented in a public health service environment with AHPs and sustained for up to 16-months with minimal external supports. External support from a more experienced researcher appeared beneficial for initially building clinician facilitator confidence and increasing internal capacity. It is important that clinicians consider their local barriers and enablers to JC implementation prior to commencing their JC, to identify key implementation strategies which are tailored to their context to support sustainability. The structured format with clear roles and expectations of members shared within the team may enable continued use of the TREAT JC format. Other supportive team factors such as a non-threatening environment for sharing, supportive managers, active participation from members and existing research and clinical knowledge may also increase sustainability. Due to changing social distancing requirements arising from the COVID-19 pandemic, JCs may need to consider an online format. Indeed, within the present study, some teams with staff across multiple locations were already using videoconferencing and anecdotally some of the continuing JCs are using the online format at the time of writing with good success.

## Conclusion

Investment in targeted interventions which can be embedded within services to help build EBP skills, confidence, and behaviours in everyday practice is vital to optimise patient outcomes [2]. This study demonstrated the value of the long-term implementation of the TREAT JC format within AHP practice settings which was able to be implemented for up to 16 monthly sessions with minimal external inputs. Moreover, this research demonstrates that at least 6 sessions may be required to meaningfully improve AHP EBP skills and confidence. While opportunities for future refinement of the TREAT format have been identified, the importance of tailoring strategies to address local barriers for long term implementation should be emphasised.

## Supplementary Information

**Additional file 1. **

**Additional file 2. **

**Additional file 3. **

**Additional file 4. **

**Additional file 5. **

## Data Availability

Please contact the corresponding author Rachel Wenke for data requests.

## Abbreviations

JC: Journal club
AHP: Allied Health Professional
EBP: Evidence-Based Practice
JCC: Journal Club Culture Questionnaire
EBPQ: Evidence Based Practice Questionnaire
