# Supplementary material for: Long term tailored implementation of structured “TREAT” journal clubs in allied health: a hybrid effectiveness-implementation study
Source: BMC Med Educ. 2022 Apr 22;22:307. doi: 10.1186/s12909-022-03333-7 (PMC9030678; doi:10.1186/s12909-022-03333-7)
Supplement: Supplementary file 1 — Additional file 1. [file 12909_2022_3333_MOESM1_ESM.docx]

| **Barrier** | **Implementation Strategy** | |
| --- | --- | --- |
| **Motivation** | |  |
| JC is not perceived to be clinically relevant | - Ensure group prioritisation of topics (consider current QI projects and clinical service priorities) | |
|  | - Longer time dedicated to discussion of application of evidence in each session | |
|  | - Provide tools regarding how to prioritise topics and integrate into practice | |
| Clinician ownership, sense of responsibility & accountability | - JC identifies 2-3 clinicians to co-facilitate JC and holder of “JC portfolio” | |
|  | - Presenting clinicians follow up action items. | |
|  | - Graduated facilitation by clinician as opposed to only research | |
| Lack of perceived benefit of format | - JC members/facilitator familiar with TREAT share positive experiences - Relate to clinical practice? – as per above | |
|  | Rotate bringing in food | |
| **Opportunity** | |  |
| Emergent leave | - Consider upskilling senior staff who do not rotate | |
| Competing demands (unprepared presenters, difficulty finding time suits everyone) | - Timetable of presenters with consistent time and place booked in clinician's calendars | |
|  | - Departmental leadership to advocate and value JC attendance and see as core business | |
|  | - Manager encouraged to attend and engage in JC as part of professional responsibility | |
|  | - “JC portfolio” holders to remind people of attendance | |
| Logistical administration (e.g., unprepared presenters, difficulty finding time) | - Email reminders prior to in calendars to prompt attendance and reduce double bookings | |
|  | - Use regular room to reduce clashes | |
|  | - JC portfolio holders to remind staff of attendance | |
| Impact of VC | - Where possible have JC face to face. | |
| Cost of food | - Rotate bringing in food | |
| Lack of participation | - Encourage group discussions during group appraisal | |
| Culture does not value EBP | - Ensure consistent message of value of EBP within actions of JC members, upon orientation to team | |
| **Capability** | |  |
| Reduced clinician confidence with EBP and journal club skills | - Attend EBP training | |
|  | - Access to online resources to assist with EBP | |
|  | - Academic mentor remains contactable and JC portfolio holders check in with mentor as needed | |
|  | - Identify clinicians who can provide support- use of modelling and support of colleagues to share knowledge | |
|  | - Having extra resources to interpret study design and assist CASP tool selection | |
|  | - Academic mentor attends initially to facilitate session then assists JC portfolio holders to facilitate using “cognitive apprentice model” | |
