# Supplementary material for: Long term tailored implementation of structured “TREAT” journal clubs in allied health: a hybrid effectiveness-implementation study
Source: BMC Med Educ. 2022 Apr 22;22:307. doi: 10.1186/s12909-022-03333-7 (PMC9030678; doi:10.1186/s12909-022-03333-7)
Supplement: Supplementary file 2 — Additional file 2. [file 12909_2022_3333_MOESM2_ESM.docx]

| **Barrier** | **Implementation Strategy**  **Implementation plan Example** | **Detail/Comments** | **By whom?** | **When?** |
| --- | --- | --- | --- | --- |
| **Motivation** | | |  |  |
| Article not perceived to be clinically relevant | - Ensure group prioritisation of topics | Every 3-4months, clinicians to email ideas for JC. Then at first 10minutes of JC, everyone votes for what topics they want for next 3-4months. | Clinician facilitator | Every 3-4 months (first will be Sept/October) |
|  | - Longer time dedicated to discussion of application of evidence in each session | Ensure that if not time to discuss that plan as an action to have a meeting outside of JC to further discuss (including senior) | Clinician facilitator to prompt actions | Each journal club (outside meetings as required) |
| **Opportunity** | | |  |  |
| Clinicians not having time to prepare | - Timetable of presenters given in advance with email reminders | Clinicians given at least 2-3 months notice of when presenting and their topic as per timetable (based on topics selected and prioritised by group) | Mary (first 3-4 topics already updated by Rachel( | Update every 2-3 months |
|  |  | Clinicians given calendar reminder given 4 weeks before and 2 weeks before they are presenting | Jane | Each month |
|  |  | Facilitator to touch base to offer support as required | month |  |
| Senior team not always in attendance | - Manager/Seniors encouraged to attend and engage in JC where able | Add to magnet feedback discussion | Jane | May 2018 |
|  |  | Feedback results of JCC survey to senior team to encourage attendance | Jack | May-June 2018 |
| VC tech issues impacting on session | - Have facilitator at times at Robina | John to facilitate from Robina where able | John | ongoing |
|  | - Ensure any VC issues are followed up promptly with IT | Facilitator to set up VC early and follow up issues as they arise | Clinician facilitator or someone they delegate to | As issues arise |
| Clinicians not reading journal beforehand | - Calendar reminders before to read article - Have articles sent out at least 1 week before | Reminder sent Tuesday before JC to read article  Clinicians aware of their topic 2months ahead and encouraged to commence search prior to so can send article well in advance | Barb  Barb (Mary to send initial emails for first two presenting topics) | Tuesday before each JC |
| **Capability** | | |  |  |
| Reduced clinician confidence with EBP and journal club skills | - Attend EBP training | Email team to raise awareness of upcoming Bond EBP training in September (Mary to attend) and Joanna Briggs training | Barb/Clinician facilitators | May/June |
|  | - Access to online resources to assist with EBP | Save useful documents in EBP folder on S drive “EBP education resources” for staff to access.  Email re: resources of STAR programme available on LOL | Sue/Clinician facilitators | May/June |
|  | - Provide incidental learning and resources during TREAT session | Have 5 min at start of session to give education about study design/ appraisal  Consider which specific areas wanting to provide upskilling in. (Through results of pre-assessment or survey monkey) | Facilitators | Ongoing  June-July |
|  | - Having extra resources to interpret study design and assist CASP tool selection and appraisal | Save useful documents in EBP folder on Shared drive “EBP education resources” for staff to access | All | Ongoing |
|  | - Academic mentor attends initially to facilitate session then assists JC portfolio holders to facilitate using “cognitive apprentice model” - Academic mentor remains contactable and JC portfolio holders check in with mentor as needed | Discussed as per Clinician facilitator guide.  Clinician facilitators to have working group as meetings re: journal club as required | All |  |
| **Other issues discussed:**   - Idea of appraising 2 articles per session (to consider in next 12 months once appraisal skills improve - Have regular meetings as JC working group- to meet again in approximately 3-4 weeks for shorter 30min meeting. | | | | |
