# Supplementary material for: Long term tailored implementation of structured “TREAT” journal clubs in allied health: a hybrid effectiveness-implementation study
Source: BMC Med Educ. 2022 Apr 22;22:307. doi: 10.1186/s12909-022-03333-7 (PMC9030678; doi:10.1186/s12909-022-03333-7)
Supplement: Supplementary file 3 — Additional file 3. [file 12909_2022_3333_MOESM3_ESM.pdf]

TREAT JOURNAL CLUB SESSION PLAN

Resources needed for session

- Minutes template for scribe
- Copies of journal article and relevant CASP checklists (also circulated by presenter prior to session)
- Snacks (optional)

Activity	Detail	Time
**Welcome**	Welcome everyone and thank their participation and those volunteering	**5 min**
**Assign Scribe**	Nominate scribe for the session (if not already allocated)	
**Review actions from last meeting**	Follow up previous actions from last meeting and troubleshoot any barriers. Modify new actions if needed (documented by scribe).	
**Clinical Question**	Clinical question/background introduced by presenting clinician(s)	**5min**
	Presenting clinician discusses process of forming PICO and search strategy (database, terms, limiters) for identifying article	
**Abstract reading & Appraisal**<br><br>(CASP)	**Everyone reads the abstract** independently (~2min)<br><br>Complete **first two questions of Section A of CASP tool together** as group and decide whether worth continuing appraisal<br><br>**Rest of Section A then appraised as a group.** For larger groups or to increase involvement, break up into groups of 2-3 and look at different questions of the tool (e.g., pairs do 1 question each and then come back to discuss after 5-10 minutes).<br><br>Incidental teaching/handouts provided as indicated.<br><br>**Discuss Sections B together**	**25 min**
**Application to context**	**Discuss section C together** (how relates to current clinical context, patient values, experience etc.), How does this influence current practice?	**15 min**
**Actions/ Follow up**	Discuss any follow up required regarding implementation of evidence and who is accountable (to be followed up at subsequent session) which is documented by the scribe.<br><br>Review topic and presenter for next meeting (brainstorm topic if none allocated).	**5min**
