# Supplementary material for: Long term tailored implementation of structured “TREAT” journal clubs in allied health: a hybrid effectiveness-implementation study
Source: BMC Med Educ. 2022 Apr 22;22:307. doi: 10.1186/s12909-022-03333-7 (PMC9030678; doi:10.1186/s12909-022-03333-7)
Supplement: Supplementary file 4 — Additional file 4. [file 12909_2022_3333_MOESM4_ESM.docx]

Supplementary File 4: Influence on Clinical Practice

| **Journal club session** | **1**  **n= (%)** | **2** | **3** | **4** | **5** | **6** | **7** | **8** | **9** | **10** | **11** | **12** | **13** | **14** | **15** | **16** | **Average/ TOTAL across sessions** |
| --- | --- | --- | --- | --- | --- | --- | --- | --- | --- | --- | --- | --- | --- | --- | --- | --- | --- |
| Attended and relevant | 20  (35.7%) | 13  (22%) | 21 (36.2%) | 20 (34.5%) | 20 (34.5%) | 23 (40.4 %) | 23 (39%) | 27 (45.7%) | 28 (48.3%) | 12 (44.4%) | 15 (33.3%) | 14 (30.4%) | 15 (32.% | 22 (47.8%) | 12 (24.5%) | 30 (65.2%) |  |
| Attended not relevant | 1 (1.8%) | 3 (5.1%) | 5 (8.6%) | 2 (3.5%) | 2 (3.5%) | 0 | 2 (3.4%) | 1 (1.69%) | 3 (5.2%) | 4 (14.8%) | 6 (13.3%) | 7 (15.2%) | 9 (19.2%) | 3 (6.5%) | 12 (24.5%) | 1 (2.2%) |  |
| Attended can’t remember | 8  (14.3%) | 7 (11.9%) | 2 (3.5%) | 5 (8.6%) | 6 (19.3%) | 4 (7.0%) | 10 (17%) | 8 (13.6%) | 8 (13.8%) | 1 (3.7%) | 5 (11.1%) | 3 (6.67%) | 10 (21.3%) | 9 (19.6%) | 7 (14.3%) | 3 (6.5%) |  |
| Did not attend | 27 (48.2%) | 35  (59.3%) | 30 (51.7%) | 31 (53.5%) | 30 (51.7%) | 30 (52.6%) | 24 (46.7%) | 23 (39%) | 19 (32.8%) | 10 (37%) | 19 (42.2) | 22 (47.8) | 13 (27.7%) | 12 (26.1%) | 18 (36.7%) | 12 (26.1%) |  |
| Average change/confirmation  Mean (SD) | 3.57 (0.57)  N=28 | 3.88  1.05) N=17 | 3.32 (1.07)  N=25 | 3.61 (0.7  N=23 | 3.65 (0.49) n=23 | 3.73 (0.78) n=26 | 3.92 (0.74) n=26 | 3.64 (0.83) n=28 | 3.47 )0.88) n=32 | 3.78 (0.80) n=14) | 3.63 (0.83) n=19 | 3.76 (0.44) n=17 | 3.90 (0.62)  N=21 | 3.29 (1.43) n=27 | 3.85 (0.90) n=13 | 4.09 (0.80) n=33 | 3.69 (0.22) |
| \| **Updated guideline,  process or pathway** \| \| --- \| | 9 (60%) | 3 (42%) | 8 (72%) | 3  (33%) | 4 (40%) | 6 (54.5%) | 1 (16.7%) | 4 (33%) | 3 (30%) | 1 (25%) | 2 (33%) | 2 (15.4%) | 4 (33.3%) | 2 (16.7%) | 4 (57.1%) | 8 (40%) | **64** |
| \| **Adopted new  treatment strategy  or resource** \| \| --- \| \|  \| | 8 (53.3%) | 4 (57.1%) | 3 (27.3%) | 9 (90%) | 7 (70%) | 6 (54.5%) | 5 (83.3%) | 6 (50%) | 6 (60%) | 3 (100%) | 1 (16.7%) | 7 (53.9) | 7 (58.3) | 5 (41.7%) | 3 (42.7%) | 8 (40%) | **88** |
| \| **Stopped current  practice due to**  **lack of evidence** \| \| --- \| | 0 | 0 | 0 | 0 | 0 | 0 | 0 | 1 (8.3%) | 0 | 0 | 0 | 0 | 0 | 0 | 0 | 0 | **1** |
| \| **Commenced**  **new research project** \| \| --- \| \|  \| | 0 | 0 | 1 (9.1%) | 0 | 0 | 0 | 0 | 2 (16.7%) | 0 | 0 | 0 | 0 | 1 (8.3%) | 0 | 1 (14.29%) | 1 (6.7%) | **6** |
| **Commenced new quality project** | 4 (26.7%) | 1 (14.3%) | 1  (9.09%) | 0 | 1 (11.1%) | 2 (18.2%) | 0 | 3 (25%) | 0 | 0 | 3 (50%) | 5 (38.5%) | 0 | 2 (16.7%) | 1 (14.3%) | 7 (36.8%) | **30** |
