# Supplementary material for: Long term tailored implementation of structured “TREAT” journal clubs in allied health: a hybrid effectiveness-implementation study
Source: BMC Med Educ. 2022 Apr 22;22:307. doi: 10.1186/s12909-022-03333-7 (PMC9030678; doi:10.1186/s12909-022-03333-7)
Supplement: Supplementary file 5 — Additional file 5. [file 12909_2022_3333_MOESM5_ESM.docx]

Supplementary File 5
Level of support provided by research mentors across JC sessions

| **Level of support provided** | | **Session**  **1** | **Session**  **2** | **Session 3** | **Session 4** | **Session 5** | **Session 6** | **Session 7** | **Session 8** | **Session 9** | **Session 10** |
| --- | --- | --- | --- | --- | --- | --- | --- | --- | --- | --- | --- |
| Maximal support    No support | Facilitated session | ⬤ ⬤ ⬤ ⬤ | ⬤ ⬤ ⬤ ⬤ | ⬤ | ⬤ | ⬤ | ⬤ | ⬤ | ⬤ |  |  |
|  | Co-facilitated with clinician |  |  | ⬤ ⬤ | ⬤ ⬤ |  |  |  |  | ⬤ ⬤ | ⬤ |
|  | Attended session as a participant and provided active support outside session |  |  | ⬤ | ⬤ | ⬤ ⬤ | ⬤ ⬤ |  | ⬤ |  |  |
|  | Did not attend session but provided active support outside of session |  |  |  |  | ⬤ |  |  |  |  |  |
|  | Attended session as a participant with NO active support outside session |  |  |  |  |  |  | ⬤ |  |  | ⬤ |
|  | Did not attend session and did not provide active support outside session |  |  |  |  |  | ⬤ | ⬤ ⬤ | ⬤ ⬤ | ⬤ | ⬤ |

Note: ⬤= Research Mentor 1 ⬤= Research Mentor 2 ⬤ = Research Mentor 3 ⬤= Research Mentor 4
